# SAPS2, APACHE2, SOFA, and Core-10-TISS upon admission as risk indicators for ICU-acquired infections: a retrospective cohort study

**DOI:** 10.1007/s15010-022-01972-y

**Published:** 2023-01-13

**Authors:** Katharina Ginter, Frank Schwab, Michael Behnke, Martin Wolkewitz, Petra Gastmeier, Christine Geffers, Friederike Maechler

**Affiliations:** 1 Institute of Hygiene and Environmental Medicine, Charité-Universitätsmedizin Berlin, Corporate Member of Freie Universität Berlin, Humboldt-Universität zu Berlin, and Berlin Institute of Health, Campus Benjamin Franklin, Hindenburgdamm 27, 12203 Berlin, Germany; 2 Institute of Medical Biometry and Statistics, Faculty of Medicine and Medical Center, University of Freiburg, Stefan Meier Str. 26, 79104 Freiburg, Germany

**Keywords:** ICU-acquired infections, Hospital-acquired infections, SAPS2, APACHE2, Intensive care unit, Severity-of-illness scoring system

## Abstract

**Purpose:**

Early identification of high-risk patients is an important component in improving infection prevention. The SAPS2, APACHE2, Core-10-TISS, and SOFA scores are already widely used to estimate mortality, morbidity and nursing workload, but this study evaluated their usefulness in assessing a patient’s risk of ICU-acquired infection.

**Methods:**

We conducted a retrospective cohort study by analyzing all patient admissions to seven ICUs at Charité Berlin, Germany in 2017 and 2018. The four scores were documented by physicians on the day of admission. The infection control staff monitored daily whether the patients experienced lower respiratory tract infections (LRTIs), urinary tract infections (UTIs), or primary blood stream infections (PBSIs). For each combination of scoring system and infection type, an adjusted Fine and Gray model was fitted.

**Results:**

We analyzed 5053 ICU admissions and observed at least one ICU-acquired infection in *N* = 253 patients (incidence density: 4.73 per 1000 days). 59.0% (*N* = 2983) of the patients were male, median age was 66 years (IQR 55–77) and median length of stay was 6 days (IQR 4–12). All models showed that patients with a higher score value were at higher risk for ICU-acquired first PBSI, LRTI, or UTI, except for the model of APACHE2 and PBSI. Patients with a SAPS2 score of > 50 points showed an increased risk of infection of sHR = 2.34 for PBSIs (CI 1.06–5.17, *p* < 0.05), sHR = 2.33 for LRTIs (1.53–2.55, *p* < 0.001) and sHR = 2.25 for UTIs (1.23–4.13, *p* < 0.01) when compared to the reference group with 0–30 points.

**Conclusions:**

The result of this study showed that admission scores of SAPS2, Core-10-TISS, APACHE2, and SOFA might be adequate indicators for assessing a patient’s risk of ICU-acquired infection.

**Supplementary Information:**

The online version contains supplementary material available at 10.1007/s15010-022-01972-y.

## Purpose

Hospital-acquired infections are a major health issue worldwide. They result in longer hospitalizations [1, 2], increased mortality of patients [3] and additional costs, which are estimated at €5800 to €11,800 per infected patient for the German public health care system [2]. Infections can be particularly serious for highly vulnerable patients in intensive care units (ICUs). Albeit infection rates have decreased in recent years [4], Suetens et al. reported a prevalence of 19.2% ICU-acquired infections in 2016/2017 [5], indicating that infections are still a common clinical condition in European ICUs.

Several authors have estimated that infection rates could be reduced by 20–55% through increased preventive measures [6–8]. Many of those have already been established in the past years, but it remains difficult to identify high-risk patients both early and easily. To address this issue, findings from surveillance data can help to identify problematic patterns and to personalize infection prevention measures, resulting in both financial and personnel savings. In this study, we focused on the analysis of admission scores of the SAPS2, APACHE2, Core-10-TISS and SOFA as indicators of an increased risk of infection. These are already used in ICUs for other reasons and would therefore be well suited for early, low-effort patient characterization.

In the past years, the relation between those four scoring systems and ICU-acquired infections has already been analyzed in different studies with different statistical approaches, yielding diverging conclusions [9–11]. The present analysis has therefore extended previous statistical approaches to an analysis of the relation between the four scoring systems SAPS2, APACHE2, Core-10-TISS, and SOFA and the three most common infection types using German ICU data.

## Methods

We conducted a retrospective cohort study by analyzing all patient admissions to four medical and three surgical ICUs at Charité University Hospital Berlin, Germany, in 2017 and 2018. All patient admissions with a length of stay of three days or more were included. Moreover, ICU admissions were only considered if all scoring parameters, i.e., SAPS2, APACHE2, SOFA and Core-10-TISS, were fully documented on the day of admission. Similarly, patients had to be older than 15 years of age, as the SAPS2 score is not suitable for pediatric patients. All patient admissions with incorrect database entries were excluded. Patients were monitored throughout their ICU stay and were considered newly admitted if absent for more than 24 h from the respective ward.

### Scoring systems

The SAPS2, APACHE2, SOFA, and Core-10-TISS scores were documented for each ICU admission by the respective physicians in charge. For all parameters, the patient’s worst recorded value within 24 h after admission was used for the calculation. A brief description of the scoring systems is given below; more details regarding the calculation are provided in Supplementary Tables 1–4, Online Resource 1.

SAPS2 (Simplified Acute Physiology Score II) The SAPS2 score is primarily used to predict patient mortality [12]. It is a weighted sum of 12 physiological parameters, the type of admission, and the presence of chronic diseases. (See Supplementary Table 1, Online Resource 1).

APACHE2 (Acute Physiology And Chronic Health Evaluation II) Similarly to SAPS2, the APACHE2 classification system is a quantification of disease severity and mortality risk. It is calculated from 12 physiological parameters, age, and chronic health points [13]. (See Supplementary Table 2, Online Resource 1).

SOFA (Sequential Organ Failure Assessment) This scoring system was developed to easily quantify patient morbidity due to sepsis. It is defined as the sum of dysfunction points of six organ systems [14, 15]. (See Supplementary Table 3, Online Resource 1).

Core-10-TISS (Simplified Therapeutic Intervention Scoring System) The Core-10-TISS approximates the nursing workload per 24 h and assigns points according to the complexity of the required procedures, whereby each unit point corresponds to 10.6 min of work [16]. The number 10 represents the shortened version, which includes 10 of the most time-consuming parameters (see Supplementary Table 4, Online Resource 1).

### Surveillance and definition of ICU-acquired infections

The occurrence of ICU-acquired infections was monitored by infection control staff using the validated protocol of the ITS-KISS Hospital Infection Surveillance System [17–19]. Pseudonymous information on the type of infection and the date of infection was recorded.

In accordance with the internationally most commonly used ECDC (European Centre for Disease Prevention and Control) and NHSN (National Healthcare Safety Network) definition of hospital-acquired infections, infections were classified as ICU-acquired, if “the onset of the signs and symptoms was on day 3 of the current admission or later […]” [20]. Accordingly, infections acquired in a regular ward before admission to intensive care were not included.

The present study examined patients with at least one of the three following types of ICU-acquired infections: primary blood stream infections (PBSIs), lower respiratory tract infections (LRTIs) and urinary tract infections (UTIs). Diagnosis was made using the ITS-KISS definitions [17] derived from the CDC criteria for hospital-acquired infections [21].

### Data collection, storage and processing

The independent variables, i.e., the scoring, as well as the dependent variables, i.e., the occurrence of infection, were documented at different points of time and in different databases, thereby ensuring blinding. For administrative reasons, 30 of the total 168 observation months (7 ICUs × 24 months of observation) had not been fully monitored for infections. If continuous infection surveillance was interrupted at any time, the patient’s stay was excluded from the complete case analysis, but was analyzed in the full data analysis. To identify potential confounders, additional information such as age, sex, ventilation on admission day, transfer from an external hospital, comorbidities, length of stay, type of ICU, and, if applicable, date of death was documented. The Ethics Committee of the Charité—Universitätsmedizin Berlin has approved this study (EA4/145/20).

### Statistical analysis

To summarize the continuous variables, the median and interquartile ranges were used.

We evaluated the infection data from both a prognostic and an etiologic perspective, hereby following the nomenclature used by Noorzdij et al. [22].

For primary analysis, we used the prognostic Fine and Gray subdistribution hazard model. The subdistribution hazard ratio (sHR) quantifies the likelihood of an individual patient acquiring an infection while in the ICU. Separate models were fitted for each scoring system, both for all ICU-acquired infections combined and for each infection type. All models were adjusted for sex, ICU type and admission type “referral from another hospital” and were accounted for the competing events “death without prior infection” and “discharge without prior infection.” For each score, one model was calculated assuming linearity, i.e., effect per one score point, and one model was calculated using score subgroups. Those subgroups were based on the quartiles of the scores, which were rounded up or down to the nearest number divisible by 5 for easier clinical applicability (with the exception of the SOFA score).

#### Sensitivity analysis

Furthermore, we investigated the relationship between the scoring systems and the infection rate from an etiologic perspective using Cox proportional hazard regression models for sensitivity analyses. They were developed to quantify causal relationships [22]. The Cox models evaluate the instantaneous infection rate of patients without previous infection during the same hospital stay. Additionally, we performed Fine and Gray models as full data analysis to avoid potential selection bias.

Descriptive and inferential statistics were calculated with R Version 3.6.3 [23] using the implementation of Fine and Gray models *crr()* from the library *cmprsk* and the implementation of Cox models *coxph()* from the library *survival.* For each type of infection, only the first ICU-acquired infection per admission was considered. The structure of the underlying database and the code used can be found in the Online Resource 2. Proportional hazards were assumed for all models. A *p* value below 0.05 was considered statistically significant. All analyses were exploratory in nature.

## Results

**Fig. 1:**
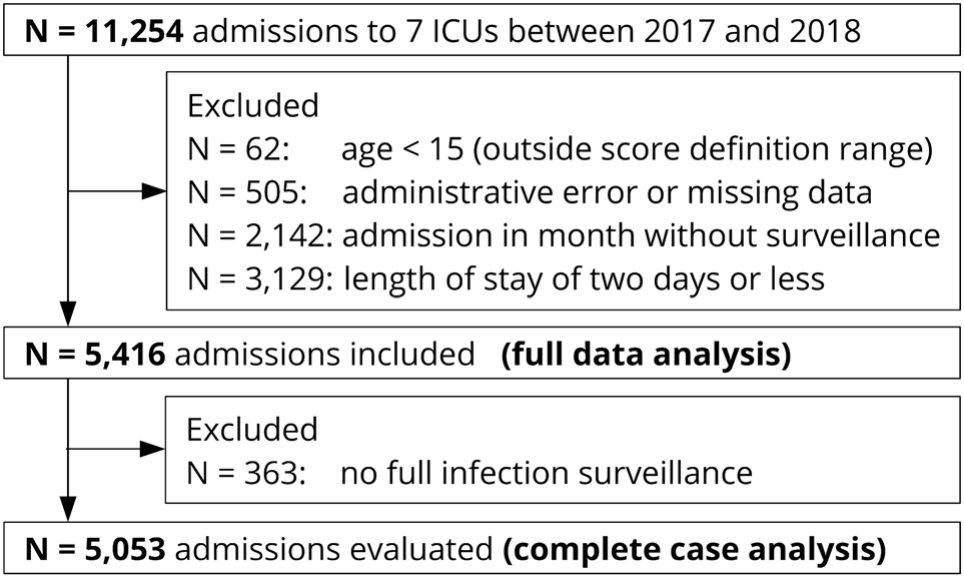
Overview of included and excluded ICU admissions. *N* number, *ICU* intensive care unit

We analyzed 5053 admissions of 4361 individual patients to seven ICUs at Charité University Hospital Berlin in 2017 and 2018. The initial dataset contained 11,254 admissions, of which a total of 5,838 were excluded due to age < 15, invalid data, admission outside the surveillance period, or a length of stay of two days or less (see Fig. 1).

Further *N* = 363 ICU admissions were excluded from the complete case analysis due to incomplete surveillance. For further descriptive information on excluded cases, please refer to Supplementary Table 5, Online Resource 3.

During the 2-year period, 53,448 patient days were examined in this study. The median duration from admission to discharge was 5 days (IQR 4–10), to death was 8 days (IQR 4–14), to first ICU-acquired infection was 11 days (IQR 6–22). A total number of 631 (12.5%) patients died during their stay on the ICU. Information on clinical characteristics is described in Table 1. More details including all events and comorbidities can be found in Supplementary Table 7, Online Resource 4.

**Table 1:** Baseline characteristics of 5,053 ICU admissions, stratified by the occurrence of ICU-acquired infections

	ICU-acquired infection = YES (*N* = 253)	ICU-acquired infection = NO (*N* = 4800)
Sex—Male	153 (60.5%)	2830 (59.0%)
Age (in years), Mdn [Q1–Q3]	68 [56–77]	67 [55–77]
Admission from external hospital	56 (22.1%)	866 (18.0%)
Deceased during ICU stay	63 (24.9%)	568 (11.8%)
Length of stay (in days), Mdn [Q1–Q3]	27 [17–43]	6 [4–11]
Ventilation on admission day	225 (88.9%)	3024 (63.0%)
Type of ICU medical (vs surgical)	113 (44.7%)	1950 (40.6%)
Scoring systems		
SAPS2, Mdn [Q1–Q3]	44 [32–61]	37 [27–51]
APACHE2, Mdn [Q1–Q3]	21 [14–27]	17 [11–24]
Core-10-TISS, Mdn [Q1–Q3]	31 [25–36]	28 [22–34]
SOFA, Mdn [Q1–Q3]	7 [4–10]	5 [2–8]
ICU-acquired infections		
Time until first ICU-AI (in days), Mdn [Q1-Q3]	11 [6–22]	
Primary blood stream infection (PBSI)	54 (21.3%)	
Lower respiratory tract infection (LRTI)	144 (56.9%)	
Urinary tract infection (UTI)	70 (27.7%)	

All values were rounded to two decimal places

*N* number, *SAPS2* Simplified Acute Physiology Score II, *Core-10-TISS* Simplified Therapeutic Intervention Scoring System, *APACHE2* Acute Physiology And Chronic Health Evaluation II, *SOFA* Sequential Organ Failure Assessment, *PBSI* primary bloodstream infection, *LRTI* lower respiratory tract infection (pneumonia and/or bronchitis), *UTI* Urinary tract infection, *Q1* first quartile, *Q3* third quartile, *Mdn* median

**Fig. 2:**
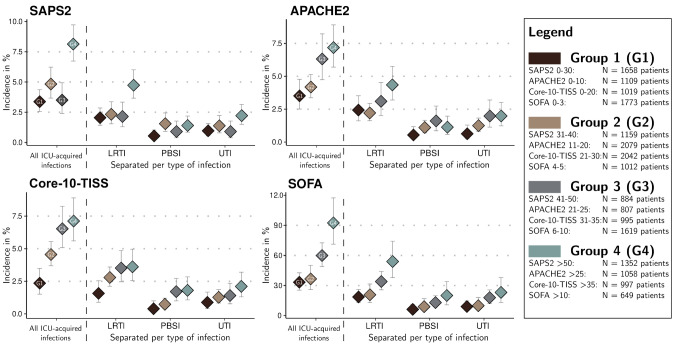
Incidence of ICU-acquired infections stratified by score group: 5053 ICU admissions at Charité Berlin. *N* number, *ICU-AI* ICU-acquired infection, *SAPS2* Simplified Acute Physiology Score II, *Core-10-TISS* Simplified Therapeutic Intervention Scoring System, *APACHE2* Acute Physiology And Chronic Health Evaluation II, *SOFA* Sequential Organ Failure Assessment, *LRTI* lower respiratory tract infection, *PBSI* primary blood stream infection, *UTI* urinary tract infection. Figure 2 shows one diagram per score examined. In each diagram, to the left of the vertical dashed line, the incidences per score group (G1–G4) are shown for all types of infections together. To the right of the vertical line, the incidences per score group are displayed (color-coded), broken down by type of infection. Groups are based on quartiles of the respective score (see methods section)

We observed at least one ICU-acquired infection in *N* = 253 patients. However, as some patients acquired two infections on ICU, the total number of infections encountered was *N* = 272. This corresponded to an overall incidence of 5.01% and an incidence density of 4.73 ICU-acquired infections per 1000 patient days (CI 4.17–5.35). Figure 2 shows increased incidences of ICU-acquired infections for higher subgroups, both for all infection types combined (on the left of the vertical dashed line) and for each infection type separately. Broken down by infection type, we detected a total of 144 patients with at least one LRTI (incidence of 2.8% or 2.69 per 1000 days), 54 patients with at least one PBSI (1.1%, 1.01 per 1000 days), and 70 patients with one or more UTI (1.4%, 1.31 per 1000 days).

**Fig. 3:**
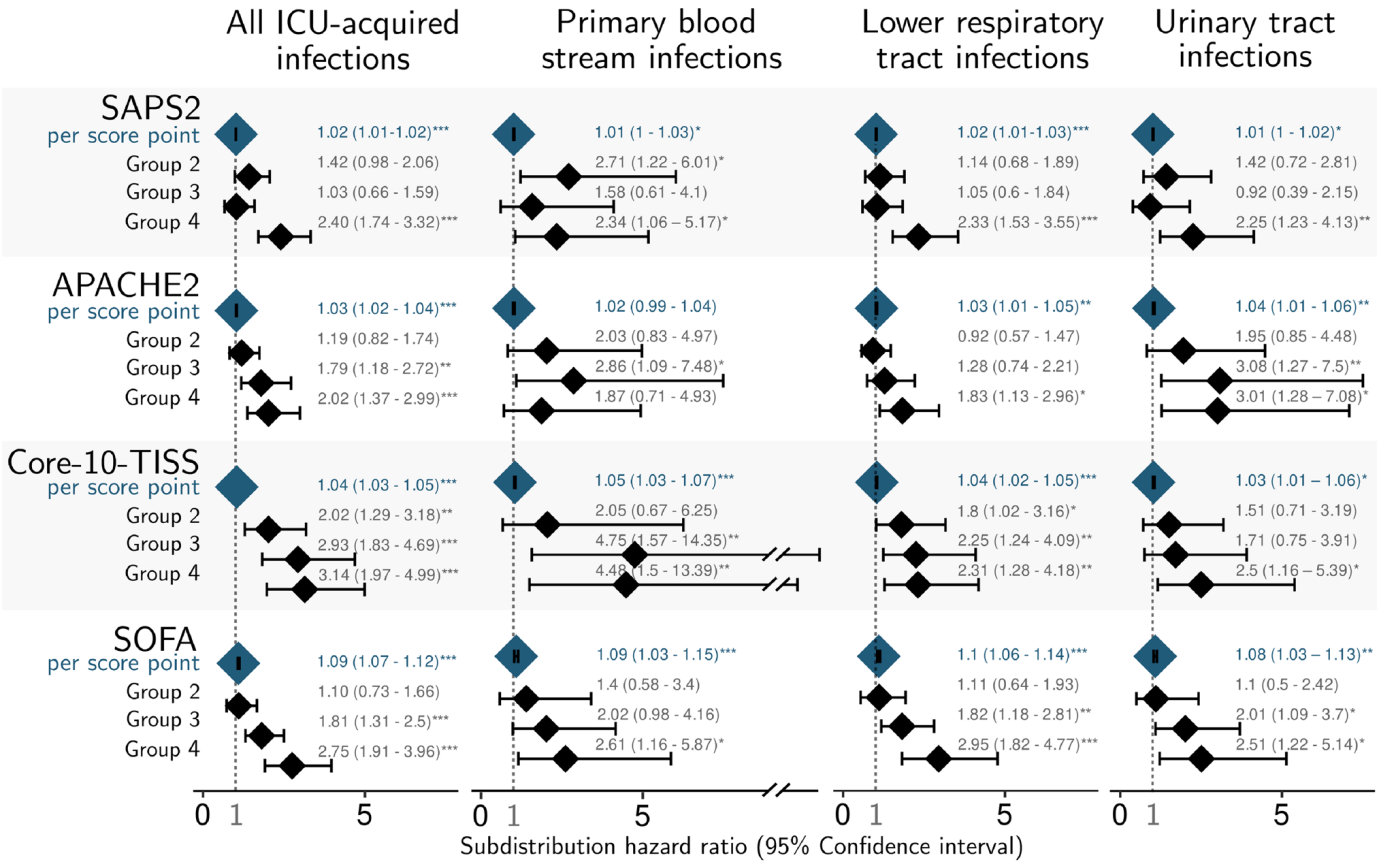
Results of the Fine and Gray models for the scores SAPS2, APACHE, SOFA and Core-10-TISS. SAPS2: reference group: 0–30, Group 2: 31–40, Group 3 41–50, Group 4 > 50, APACHE2: reference group: 0–10, Group 2: 11–20, Group 3: 21–25, Group 4 > 25, Core-10-TISS: reference group 0–20, Group 2: 21–30, Group 3: 31–35, Group 4: > 35, SOFA: reference group 0–1, Group 2: 4–5, Group 3: 6–10, Group 4 > 10, SAPS2, Simplified Acute Physiology Score II; Core-10-TISS, Simplified Therapeutic Intervention Scoring System; APACHE2, Acute Physiology And Chronic Health Evaluation II; SOFA, Sequential Organ Failure Assessment. Significance level: ***< 0.001, **< 0.01, *< 0.05. All models are adjusted by sex, admission from external hospital, and ICU type. Each score was evaluated once as a continuous variable (“per score point,” first row) and grouped

The results of the main analysis for the endpoints of the ICU-acquired infections are shown in Fig. 3. Shortly summarized, for almost all combinations of scores and infection types, the models revealed that a higher score value was associated with an increased risk of ICU-acquired infection, except for APACHE2 and PBSI. In addition, the risk of in-hospital death increased, while the likelihood of discharge decreased accordingly (data not shown). When examining the grouped scores, the highly increased daily risk of infection of the patients with the highest Core-10-TISS score subgroup (sHR = 3.14, CI 1.97–4.99) is noticeable when compared to SAPS2 (sHR = 2.4), APACHE2 (sHR = 2.02), and SOFA (sHR = 2.75). This can be explained by the increased daily risk for PBSI of sHR = 4.48 (CI 1.5–13.99), while the sHR for LRTI and UTI ranged between 2 and 3, similar to the other scores. When evaluating the scores as continuous variables, the daily risk of any first ICU-acquired infection increases by 2% (CI 1–3%) per SAPS2 point (range 0–111), by 3% (CI 1–5%) per APACHE2 point (range 0–56), by 4% (CI 2–5%) per Core-10-TISS point (range 6–68) and even by 9% (CI 6–14%) per SOFA point (range 0–22). It should be noted, however, that this is mainly due to the fact that scores with a smaller range of values are expected to have a higher effect per score point than scores with a large range of values.

Additionally, we performed several sensitivity analyses. In contrast to the main analysis, the etiologic event-specific Cox models showed no association between the SAPS2, APACHE2, Core-10-TISS, or SOFA score and the conditional daily rate of ICU-acquired PBSI, LRTI, or UTI. However, with regard to the increased daily mortality rate and the decreased daily rate of discharge at higher score levels, the models were able to confirm the results of the primary analysis (see Supplementary Table 8, Online Resource 5). Additionally, a full data analysis was performed, including all months without continuous infection surveillance. A total of 5,398 admissions with 286 ICU-acquired infections (incidence 5.3%) were evaluated (see Supplementary Table 5, Online Resource 3). Among other differences, the drop-outs stayed longer on ICU (22 days vs 6 days, *p* < 0.01), were more often ventilated (81,5% vs 64,3%, *p* < 0.01) and had a higher incidence of ICU-acquired infections (9.1% vs 5.0%, *p* < 0.01) (see Supplementary Table 5, Online Resource 3). While we observed no difference in the frequency of some important comorbidities such as diabetes mellitus or chronic lung disease, we found a more frequent occurrence of peptic ulcers (3.3% vs 4.4%, *p* = 0.035) and cerebrovascular diseases (18.0% vs 22.0% *p* < 0.01), but less cardiovascular diseases (50.5% vs 28.7%, *p* = 0.01). Nevertheless, in accordance with the results from the primary analysis, the full data analysis revealed that an elevated SAPS2, APACHE2, Core-10-TISS, and SOFA was associated with a higher daily risk of infection (see Supplementary Table 6, Online Resource 3).

## Discussion

The present study suggests that patients with higher SAPS2, APACHE2, Core-10-TISS, and SOFA scores have both a higher risk of ICU-acquired infection and in-hospital death, as well as a lower probability of discharge.

Our results may feel intuitive and could be explained as follows: The four scouring system quantifies a patient’s disease severity, which could impact the occurrence of both endogenous and exogenous ICU-acquired infections. In intrinsic or endogenous infections, the pathogens originate from normally harmless commensal skin flora and can lead to infection if the immune system is weakened, e.g., due to high disease severity, which is then depicted in a higher score. Exogenous infections, also called cross-infections, originate through transmission of pathogens from sources outside the patient body, such as contaminated hospital equipment, other patients or hospital staff. Critically ill patients generally require more interventions and devices, so disease severity could also be associated with a higher risk of acquiring exogenous infections.

Several factors may account for the fact that we observed no effect regarding the risk of infection when the reference group, which included the admissions with the lowest score values, was compared with the subgroup with the second-lowest score values: First, the scores might have a low impact on the risk of infection in this group. Second, the risk of infection might only increase above a certain threshold value. And third, even though we investigated a relatively large study population, the sample size may not have been sufficient to show the differences in each of the subgroups.

The literature is poor on studies using similar statistical methods, known as time-to-event-analyses, to examine the relationship between established scoring systems and the occurrence of ICU-acquired infections. Some of those studies described the APACHE2 score as a risk factor for ICU-acquired infections [9, 10], whereas others found no such association [11]. All studies included fewer than 1000 patients; some of them specifically examined patients after surgery [10] or trauma [11]. Moreover, the three types of infections—UTI, LRTI, and PBSI—each have different symptomatology and pathogenesis and therefore require separate rather than cumulative statistical analysis. To date, however, only a few other studies of this kind have been published. One study confirmed an effect of the APACHE2 score analyzed in groups on nosocomial bacteriemia [24], but other authors have not found any effect of the SAPS2 score per score point on (ventilator-associated) pneumonia [25, 26]. This could be explained by the smaller number of patients in those two publications (761 and 1876 patients, respectively) and their adjustment for intubation [25, 26] and invasive positive pressure ventilation in [25]. To our knowledge, there are currently no comparable studies on ICU-acquired UTIs and the Core-10-TISS and SOFA scores. It should be noted that all studies published to date differ in their methodology, which could partly explain the divergent results.

Another noteworthy perspective on the four scores SAPS2, APACHE2, Core-10-TISS, and SOFA is the comparison with scores explicitly designed for hospital-acquired infections. For example, Chang et al. developed a 7-item scoring system that accounts for catheter use and specific medication, including glucocorticosteroids and prophylaxis of stress ulcers [27]. Thus, the design of their score differs from SAPS2, APACHE2, Core-10-TISS, and SOFA, which focus on physiological parameters and chronic disease variables. As mentioned previously, the latter have the advantage of already being widely established on ICUs. It seems questionable whether a clinical implementation of the 7-item scoring system by Chang et al. would be feasible as the recording of parameters requires additional time and effort for physicians and nursing staff. In addition, the use of their score is limited by the fact that certain parameters such as medication and catheter use usually cannot be determined on the first day in the ICU, which could complicate early identification of high-risk patients. These limitations rather stress the benefits of using established admission scores.

This study is subject to several limitations. In the present study, we used the methods of the ITS-KISS Hospital Infection Surveillance System to identify ICU-acquired infections. It offers the advantage of validated protocols, quality assurance and a standardized data entry system. Our observed incidence of 5.01% ICU-acquired infections was comparatively low. This could be due to our focus on three types of infections (PBSI, UTI, LRTI) and the exclusion of all infections prior to a patient’s transfer to the ICU. In addition, there was the possibility of underreporting by infection control staff or incorrect database entries. For administrative reasons, there were months without monitoring. We therefore performed an additional full data analysis and were able to confirm the results from the primary analysis (see Supplementary Table 6, Online Resource 3). In addition, the external validity of this study might be limited by its design as a single-center study. Thus, multicenter studies involving different types of hospitals with different germ spectra might be useful for further investigation.

When performing statistical analyses with infection data, competing risks such as death and discharge must be adequately considered due to their frequent occurrence. The risk of an ICU-acquired infection depends on the occurrence of the competing events: By definition, an infection cannot occur in a patient who is deceased or has already been discharged. The results of the infection analysis should therefore always be seen in the context of the analysis of morbidity and discharge. In our study, we showed that the cumulative risk of infection (Fine and Gray models) increases with a higher score, but the rate of infection (Cox models) does not. This is because as the score increases, the probability of a patient being discharged without prior death or infection decreases significantly. Conversely, this leads to a higher length of stay and thus an overall higher cumulative risk of infection, while the daily infection rate may remain the same. The Fine and Gray models used in the main analysis are therefore more suitable for prognostic statements in the presence of competing risks [28].

This study aimed to contribute to an improved infection prevention in intensive care units. The novelty of our study is the separate analysis of the three infection types in a larger number of patients compared to previous studies [29]. In addition, by using the prognostic Fine and Gray model, we were able to ensure an adequate consideration of the competing events. We demonstrated that the scores are an indicator of a patient’s individual risk for ICU-acquired infections, which could allow early initiation of preventive measures in high-risk patients in daily clinical practice. In the sense of personalized medicine, preventive measures could thus be specifically adapted to the individual patient in order to use existing human and financial resources as effectively as possible. They could be used to identify patients which benefit most from interventions: For example, antiseptic washings lead to a reduction in hospital-acquired infections on the one hand [30], but might increase the likelihood of resistance development and lead to alteration of the microbiome [31] on the other. The scores evaluated here could therefore be a useful aid in selecting patients at high risk. Moreover, future studies could evaluate the use of the scores as decision support tool by additionally analyzing daily scores throughout a patient’s ICU stay. This approach could also take into account the patient’s response to treatment and disease progression, yet a major drawback would be the significantly higher documentation requirements on the respective ward. Equally helpful could be further refinements of the predictive power of the scores, e.g., through the use of clinically informed score thresholds in light of the nonlinear increase in infection risk, or through a combination of the four established scores or their parameters.

## Conclusions

In summary, in this study, we evaluated the usefulness of four scoring systems, SAPS2, APACHE2, Core-10-TISS, and SOFA as risk indicators for ICU-acquired infections. Originally, they were designed for other outcomes, such as mortality, morbidity and nursing workload. The results of our study showed that the admission values of the four scoring systems might be good predictors for the ICU-acquired infection risk of a patient and could be used for the early detection of high-risk patients in clinical practice.

## Supplementary Information

Below is the link to the electronic supplementary material.

Calculation Tables for SAPS2, APACHE2, Core-10-TISS, SOFA (PDF 69 KB)

R Script Code for Cox Models and Fine-and-Gray Models (PDF 33 KB)

Results of full data analysis: descriptive table and results of Fine and Gray models (PDF 49 KB)

Baseline characteristics of 5,053 ICU admissions, stratified by the occurrence of an ICU-acquired infection and all competing events (PDF 42 KB)

Results of the cause-specific Cox models for the scores SAPS2, APACHE, SOFA and Core-10-TISS (PDF 53 KB)

## Data Availability

Data for intensive care units are available from the Data Access Committee of the German National Reference Center for Surveillance of Nosocomial Infections for researchers who meet the criteria for access to confidential data and agree to be bound by a non-disclosure agreement regarding the publication of unaggregated data. The data are collected according to German law and the National Reference Center for Surveillance of Nosocomial Infections is bound by a non-disclosure agreement with the hospitals participating in the system. We are not allowed to publish unaggregated data that might lead to identification of individual hospitals or intensive care units directly or by third parties. Researchers wishing to obtain the data set required to reproduce our findings are asked to direct their request to: Data Access Committee Nationales Referenzzentrum (NRZ) für Surveillance von nosokomialen Infektionen am Institut fuer Hygiene und Umweltmedizin Charité-Universitätsmedizin Berlin (nrz@charite.de).

## Abbreviations

ICU: Intensive care unit
ITS-KISS: “Intensivstation—Krankenhaus Infektions Surveillance System” = German ICU-Hospital Infection Surveillance System
LRTI: Lower respiratory tract infection
PBSI: Primary blood stream infection
UTI: Urinary tract infection
sHR: Subdistribution hazard ratio
CI: Confidence interval
SAPS2: Simplified Acute Physiology Score II
APACHE2: Acute Physiology And Chronic Health Evaluation II
Core-10-TISS: Simplified Therapeutic Intervention Scoring System (including 10 core items)
SOFA: Sequential Organ Failure Assessment
