# Supplementary material for: SAPS2, APACHE2, SOFA, and Core-10-TISS upon admission as risk indicators for ICU-acquired infections: a retrospective cohort study
Source: Infection. 2023 Jan 13;51(4):993–1001. doi: 10.1007/s15010-022-01972-y (PMC10352405; doi:10.1007/s15010-022-01972-y)
Supplement: Supplementary file 1 — Calculation Tables for SAPS2, APACHE2, Core-10-TISS, SOFA (PDF 69 KB) [file 15010_2022_1972_MOESM1_ESM.pdf]

SAPS2, APACHE2, SOFA, and Core-10-TISS upon admission as risk indicators for ICU-acquired infections: a retrospective cohort study

published in: Infection

Authors: Katharina Ginter[1], Frank Schwab[1], Michael Behnke[1], Martin Wolkewitz[2], Petra Gastmeier[1], Christine Geffers[1,3] and Friederike Maechler[1,3]*

[1]Institute of Hygiene and Environmental Medicine, Charité - Universitätsmedizin Berlin, corporate member of Freie Universität Berlin, Humboldt-Universität zu Berlin, and Berlin Institute of Health, Campus Benjamin Franklin, Hindenburgdamm 27, 12203 Berlin, Germany

[2] Institute of Medical Biometry and Statistics, Faculty of Medicine and Medical Center, University of Freiburg, Stefan Meier Str. 26, 79104 Freiburg, Germany

[*]corresponding author, correspondence: friederike.maechler@charite.de

[3]these authors contributed equally and share last authorship

Additional File 1: Calculation Tables for SAPS2, APACHE2, Core-10-TISS, SOFA

Supplementary Table 1: SAPS2 (without Glasgow Coma Scale) – Calculation Table

Variable / points:	0	1	2	3	4	5	6	7	8	9	10	11	12	13	14	15	16	17	18
**Age, y**	<40							40-59					60-69			70-74	75-79		≥80
**Heart rate, beats/min**	70-119		40-69		120-159			≥160				<40							
**Systolic blood pressure, mmHg**	100-199		≥200			70-99								<70					
**Body temperature, °C**	<39		≥39																
**PaO_2 mmHg/FiO_2***							≥200			100-199		<100							
**PaO_2, kPa/FiO_2***							≥26.6			13.3-26.5		<13.3							
**Urinary output, L/d**	≥1.000				0.500-0.999							<0.500							
**Serum urea level, mmol/L (g/L) or serum urea nitrogen level, mg/dL**	<10 (<0.60) <28						10.0-29.9 (0.60-1.79) 28-83				≥30.0 (≥1.80) ≥84								
**White Blood Count count (10^3/cu mm)**	1.0-19.9			≥20.0							<1.0								

**Serum potassium, mmol/d**	3.0-4.9			<3.0<br>≥5.0															
**Serum sodium level, mmol/L**	125-144	≥145				<125													
**Serum bicarbonate level, mEq/L**	≥20			15-19			<15												
**Bilirubin level, µmol/L (mg/dL)**	<68.4 (<4.0)				68.4-102.5 (4.0-5.9)					≥ 102.6 (≥6.0)									
**Glasgow Coma Scale**	5 points: 11-13<br>7 points: 9-10<br>13 points: 6-8<br>26 points: <6																		
**Chronic diseases**	9 points: metastatic canter<br>10 points: hematologic malignancy<br>17 points: AIDS																		
**Type of admission**	0 points: scheduled surgical<br>6 points: Medical<br>8 points: Unscheduled surgical																		

* Only if ventilated or continuous positive airway pressure

Sources:
(1) Le Gall, J. R., S. Lemeshow, and F. Saulnier, 'A New Simplified Acute Physiology Score (SAPS II) Based on a European/North American Multicenter Study', JAMA, 270.24 (1993), 2957–63 <https://doi.org/10.1001/jama.270.24.2957>
(2) 'Errors in Tables.', JAMA: The Journal of the American Medical Association, 271.17 (1994), 1321 <https://doi.org/10.1001/jama.1994.03510410033028>

Supplementary Table 2: APACHE2 – Calculation Table

**variable / points**	**0**	**1**	**2**	**3**	**4**	**5**	**6**
**A Acute Physiology Score (APS) Points: Sum of the 12 individual variable points**							
1 Temperature – rectal (°C)	36-28.4	18.5-38.9<br>24-35.9	32-33.9	39-40.9<br>30-31.0	≥ 41<br>≤ 29.9		
2 Mean arterial pressure - mmHg	70-109		110-129<br>50-60	130-159	≥ 160<br>≤ 49		
3 Heart rate in beats per minute	70 - 139		110- 139<br>55-69	140 – 179<br>40-54	≥ 180<br>≤ 39		
4 Respiratory Rate (non ventilated or ventilated) breaths/min	12-24	25-34<br>10-11	6-9	35-49	≥ 50<br>≤5		
5 Oxygenation (mmHg)<br>if FiO2 ≥ 0.5: A-aDO$_2$<br>if FiO$_2$ <0.5: PaO$_2$	<200<br>>70	61-70	200-349	150-499<br>55-60	>500<br><55		
6 Arterial pH	7.33-7.49	7.5-7.59	7.25-7.32	7.6 - 7.69<br>7.15 – 7.24	≥ 7.7<br>< 7.15		
If no ABG: Serum HCO$_3$ (venous, mMol/L)	22-31.9	32-40.9	18-21.9	41-51.9<br>15-17.9	≥ 52<br><15		

7 Serum sodium (mMol/L)	130-149	150-154	155-159<br>120-129	160-179<br>111-119	≥ 180<br>≤ 110		
8 Serum potassium (mMol/L)	2.5-5.4	5-5.9<br>3-3.4	 2.5-2.9	6-6.9	≥ 7<br><2.5		
9 Serum creatinine (mg/100ml) [b]	0.6-1.4		1.5-1.9<br><0.6	2-3.4	≥ 3.5		
10 Hematocrit (%)	30-45.9	46-49.9	50-59.9<br>20-29.9		≥ 60<br><20		
11 White Blood Count (total/mm^3) in 1,000s)	3-14.9	15-19.9	20-39.9<br>1-2.9		≥ 40<br><1		
12 Glasgow Coma Scale (Score = 15 minus actual GCS) (0-12 points)							
**B Age points**							
Age	≤ 44		45-54			65-74	≥ 75
**C Chronic Health Points**							
	No chronic organ insufficiency or immuno-compromise history		Elective postoperative patients [a]			For non-operative or emergency post-operative patients [a]	

[a] and chronic organ insufficiency or immunocompromise history: must have been evident prior to this hospital admission and conform the following criteria

- liver: biopsy proven cirrhosis and documented portal hypertension; episodes of past upper GI bleeding attributed to portal hypertension; or prior episodes of hepatic failure(encephalopathy/coma)
- cardiovascular: New York Heart Association Class IV
- respiratory: Chronic restrictive, obstructive or vascular disease resulting in severe exercise restrictions, i.e., unable to climb stairs or perform household duties; or documented chronic hypoxia, hypercapnia, secondary polycythemia, severe pulmonary hypertension (>40mmHg), or respirator dependency.
- renal: Receiving chronic dialysis
- immuno-compromised: the patient has received therapy that suppresses resistance to infection, e.g. immuno-suppression, chemotherapy, radiation, long term or recent high dose steroids, or has a disease that is sufficiently advanced to suppress resistance to infection (e.g. leukemia, lymphoma, AIDS.)

[b] Double point score for acute renal failure

ABG = arterial blood gas analysis

Source: Knaus, W. A., E. A. Draper, D. P. Wagner, and J. E. Zimmerman. 1985. "APACHE II: A Severity of Disease Classification System." Critical Care Medicine 13(10): 818–29.

Supplementary Table 3: SOFA – Calculation Table

		SOFA points			
**Organ System**	**Parameter**	**1**	**2**	**3**	**4**
**Respiration**	PaO_2/FiO_2, mmHg	<400	<300	<200[a]	<100[a]
**Coagulation**	Platelets x10^3	<150	<100	<50	<20
**Liver**	Bilirubin, mg/dl (µmol/l)	1.2-1.9 (20-31)	2.0 – 5.9 (33-101)	6.0 – 11.9 (102 – 204)	> 12.0 (>204)
**Cardiovascular**	Hypotension	MAP < 70mmHg	Dopamine ≤ 5<br>or dobutamine (any dose)[b]	Dopamine > 5<br>or epinephrine ≤ 0.1<br>or norepinephrine ≤ 0.1	Dopamine > 15<br>or epinephrine > 0.1<br>or norepinephrine > 0.1
**Central nervous system**	Glasgow Coma Scale	13-14	10-12	6-9	<6
**Renal**	Creatinine, mg/dl (µmol/l) or urine output	1.2 -1.9<br>(110-170)	2.0-3.4 (171-299)	3.5 – 4.9 (300 – 440) or <500ml/day	>5.0 (>440= or <200ml/day

A with respiratory support
B Adrenergic agents administered for at least 1h (doses given are in µg/kg*min)
Note: A spelling error in the original publication was corrected: ">204" instead of "<204".

Source: J. -L. Vincent and others, 'The SOFA (Sepsis-Related Organ Failure Assessment) Score to Describe Organ Dysfunction/Failure', Intensive Care Medicine, 22.7 (1996), 707–10 <https://doi.org/10.1007/BF01709751>.

Supplementary Table 4: Core-10-TISS – Calculation Table

Ventilatory Support	
Mechanical ventilation. Any form of mechanical or assisted ventilation with or without positive end-expiratory pressure; with or without muscle relaxants; spontaneous breathing with positive end-expiratory pressure)	5
Cardiovascular Support	
Multiple vasoactive medications. More than one vasoactive drug, disregard type and dose.	4
Intravenous replacement of large fluid losses. Fluid replacement > 5 liters per square meter per day, disregard type of fluid administered.	4
Peripheral arterial catheter.	5
Left atrium monitoring. Pulmonary artery flotation catheter with or without cardiac output measurement.	8
Renal Support	
Hemofiltration techniques. Dialytic techniques.	3
Neurologic Support	
Measurement of intracranial pressure.	4
Metabolic Support	
Treatment of complicated metabolic acidosis/alkalosis.	4
Specific Interventions	
Specific interventions in the ICU. Naso or orotracheal intubation, introduction of a pacemaker, cardioversion, endoscopies, emergency surgery in the past 24 hours, gastric lavage. Routine interventions without consequences to the clinical condition of the patient, such as radiographs, echography, EKG, dressings or introduction of venous or arterial catheters, are not included.	5
Specific interventions outside of ICU. Surgery or diagnostic procedures.	5

Sources:
[1] D. R. Miranda, A. de Rijk, and W. Schaufeli, 'Simplified Therapeutic Intervention Scoring System: The TISS-28 Items--Results from a Multicenter Study', Critical Care Medicine, 24.1 (1996), 64–73 <https://doi.org/10.1097/00003246-199601000-00012>.
[2] Deutsches Institut für Medizinische Dokumentation und Information, https://www.dimdi.de/static/de/klassifikationen/ops/kode-suche/opshtml2018/zusatz-06-anh-aufwandspunkte-intensivmedizin-erwachsene.htm, accessed Dec 10, 2022
