# Supplementary material for: SAPS2, APACHE2, SOFA, and Core-10-TISS upon admission as risk indicators for ICU-acquired infections: a retrospective cohort study
Source: Infection. 2023 Jan 13;51(4):993–1001. doi: 10.1007/s15010-022-01972-y (PMC10352405; doi:10.1007/s15010-022-01972-y)
Supplement: Supplementary file 2 — R Script Code for Cox Models and Fine-and-Gray Models (PDF 33 KB) [file 15010_2022_1972_MOESM2_ESM.pdf]

SAPS2, APACHE2, SOFA, and Core-10-TISS upon admission as risk indicators for ICU-acquired infections: a retrospective cohort study

published in: Infection

*Authors: Katharina Ginter[1], Frank Schwab[1], Michael Behnke[1], Martin Wolkewitz[2], Petra Gastmeier[1], Christine Geffers[1,3] and Friederike Maechler[1,3*]*

*[1]Institute of Hygiene and Environmental Medicine, Charité - Universitätsmedizin Berlin, corporate member of Freie Universität Berlin, Humboldt-Universität zu Berlin, and Berlin Institute of Health, Campus Benjamin Franklin, Hindenburgdamm 27, 12203 Berlin, Germany*

*[2] Institute of Medical Biometry and Statistics, Faculty of Medicine and Medical Center, University of Freiburg, Stefan Meier Str. 26, 79104 Freiburg, Germany*

*[*]corresponding author, correspondence: friederike.maechler@charite.de*

*[3]these authors contributed equally and share last authorship*

# Additional File 2: R Script Code for Cox Models and Fine-and-Gray Models

The calculation of the models was based on a table with the following structure. Depending on the endpoint, the coding of "NI_endpoint_code" and “NI_time” was changed so that the first event was always evaluated.

| NI_time (time until first event) | NI_endpoint_code (0 for discharge, 1 for infection, 2 for death) | Score (SAPS2/ APACHE2/ Core-10-TISS/ SOFA) | Other variables (Sex, external admission [yes/no], ward type [medical/ surgical]) |
|---|---|---|---|
| xxx | xxx | xxx | xxx |
| xxx | xxx | xxx | xxx |

## 1 Fine-and-Gray models

```
covariable_matrix_score <- model.matrix(NI_endpoint_code ~ score + sex
    + external_admission + ward_type, data = mydata)
covariable_matrix_score = covariable_matrix_score[,-1]
fgmodel <- crr(ftime = NI_time, fstatus = NI_endpoint_code, cov1 =
covariables_matrix_score, failcode=”name_of_endpoint”)
```

Depending on the model, the string "name_of_endpoint" was replaced with the string "0", "1" or "2" respectively.

## 2 Sensitivity analysis: Cox regression Models

```
surv_d <- Surv(NI_time, NI_endpoint_code)
cox_1 <- coxph(surv_d~ score +$sex + external_admission + ward_type, data=mydata)
```

Depending on the model, NI_endpoint_code was replaced with the dichotomous (yes/no or 0/1) endpoint for infection, death, discharge, sepsis, PBSI, LRT, and UTI.
