# Supplementary material for: SAPS2, APACHE2, SOFA, and Core-10-TISS upon admission as risk indicators for ICU-acquired infections: a retrospective cohort study
Source: Infection. 2023 Jan 13;51(4):993–1001. doi: 10.1007/s15010-022-01972-y (PMC10352405; doi:10.1007/s15010-022-01972-y)
Supplement: Supplementary file 3 — Results of full data analysis: descriptive table and results of Fine and Gray models (PDF 49 KB) [file 15010_2022_1972_MOESM3_ESM.pdf]

SAPS2, APACHE2, SOFA, and Core-10-TISS upon admission as risk indicators for ICU-acquired infections: a retrospective cohort study

published in: Infection

Authors: Katharina Ginter[1], Frank Schwab[1], Michael Behnke[1], Martin Wolkewitz[2], Petra Gastmeier[1], Christine Geffers[1,3] and Friederike Maechler[1,3]*

[1]*Institute of Hygiene and Environmental Medicine, Charité - Universitätsmedizin Berlin, corporate member of Freie Universität Berlin, Humboldt-Universität zu Berlin, and Berlin Institute of Health, Campus Benjamin Franklin, Hindenburgdamm 27, 12203 Berlin, Germany*

[2] *Institute of Medical Biometry and Statistics, Faculty of Medicine and Medical Center, University of Freiburg, Stefan Meier Str. 26, 79104 Freiburg, Germany*

[*]*corresponding author, correspondence: friederike.maechler@charite.de*

[3]*these authors contributed equally and share last authorship*

Additional File 3: Results of full data analysis: descriptive table and results of Fine and Gray models

Supplementary Table 5: Baseline characteristics of 363 admissions dropped out during the study period between 2017 and 2018

	Per Protocol N = 5,053	Drop-out N = 363	P-value
Sex - Male	2,983 (59.0%)	216 (59.5%)	0.90
Age (in years), Mdn [Q1-Q3]	67.0 (55.0 - 77.0)	67.0 (56.0 - 77.0)	0.62
Admission from external hospital	4,131 (81.8%)	266 (73.3%)	< 0.0001
Deceased during ICU stay	4,422 (87.5%)	294 (81.0%)	0.0005
Length of stay (in days), Mdn [Q1-Q3]	6.0 (4.0 - 12.0)	22.0 (10.0 - 43.0)	< 0.0001
Ventilation on admission day	3,249 (64.3%)	296 (81.5%)	< 0.0001
Type of ICU medical (vs surgical)	2,063 (40.8%)	55 (15.2%)	< 0.0001
**Scoring Systems**			
SAPS2, Mdn [Q1-Q3]	38.0 (27.0 - 52.0)	42.0 (30.0 - 57.0)	< 0.0001
APACHE2, Mdn [Q1-Q3]	17.0 (11.0 - 24.0)	19.0 (13.0 - 26.0)	0.0007
Core-10-TISS, Mdn [Q1-Q3]	28.0 (22.0 - 34.0)	32.0 (27.0 - 38.0)	< 0.0001
SOFA, Mdn [Q1-Q3]	5.0 (3.0 - 8.0)	6.0 (3.0 - 9.0)	0.0002
**ICU-acquired infections**			
Any first ICU-AI	253 (5.0%)	33 (9.1%)	0.001
First primary blood stream infection (PBSI)	54 (1.1%)	11 (3.0%)	0.002
First lower respiratory tract infection (LRTI)	144 (2.8%)	18 (5.0%)	0.034
First urinary tract infection (UTI)	70 (1.4%)	8 (2.2%)	0.30
**Comorbidities / Charlson Comorbidity Index (CCI)**			
Missing	519 (10.3%)	116 (32.0%)	
CCI, Mdn [Q1-Q3]	6.0 (4.0 - 9.0)	6.0 (4.0 - 9.0)	0.82
Cardiovascular disease	1,543 (30.5%)	104 (28.7%)	0.011
Cerebrovascular disease	911 (18.0%)	80 (22.0%)	< 0.0001
Dementia	217 (4.3%)	9 (2.5%)	0.50
Chronic lung disease	979 (19.4%)	62 (17.1%)	0.22
Rheumatism	117 (2.3%)	3 (0.8%)	0.26
Peptic ulcer	165 (3.3%)	16 (4.4%)	0.035
Liver disease	649 (12.8%)	38 (10.5%)	0.71
Diabetes mellitus	1,108 (21.9%)	56 (15.4%)	0.58
Renal disease	2,132 (42.2%)	118 (32.5%)	0.87
Neoplastic disease	1,406 (27.8%)	53 (14.6%)	0.002
Hemiplegia	570 (11.3%)	48 (13.2%)	0.002
AIDS/HIV infection	11 (0.2%)	0 (0.0%)	0.93
Any infection (CCI)	3,041 (60.2%)	195 (53.7%)	0.0001

	Per Protocol N = 5,053	Drop-out N = 363	P-value
Sex - Male	2,983 (59.0%)	216 (59.5%)	0.90
Sepsis (CCI)	1,611 (31.9%)	133 (36.6%)	< 0.0001
Pneumonia (CCI)	1,607 (31.8%)	126 (34.7%)	< 0.0001

Legend:

The values were rounded to one decimal place. Continuous variables were evaluated with the Wilcoxon Rank test and proportional variables with the Chi2 test.
N, Number; SAPS2, Simplified Acute Physiology Score II; Core-10-TISS, Simplified Therapeutic Intervention Scoring System; APACHE2, Acute Physiology And Chronic Health Evaluation II; SOFA, Sequential Organ Failure Assessment; AIDS, Acquired immune deficiency syndrome; HIV, Human immunodeficiency virus; PBSI, Primary bloodstream infection; LRTI, Lower respiratory tract infection (pneumonia and/or bronchitis); UTI, Urinary tract infection; Q1, First Quartile; Q3, Third Quartile; Mdn, Median

**Combined variable:*
- cerebrovascular disease: myocardial infarction, congestive heart failure, peripheral vascular disease
- liver disease: mild liver disease, moderate to severe liver disease
- diabetes mellitus = diabetes mellitus without complication, diabetes mellitus with chronic complications
- neoplastic disease: cancer, metastatic cancer, leukemia, lymphoma

Supplementary Table 6: Full data analysis: Results of the Fine and Gray subdistribution hazard model for the scores SAPS2, APACHE, SOFA and Core-10-TISS; 5,416 ICU admissions at Charité- University Hospital Berlin between 2017 and 2018

		**Death** [sHR (95% CI)]	**Discharge** [sHR (95% CI)]	**ICU-acquired infection** [sHR (95% CI)]	**ICU-acquired infection: Type**		
					**PBSI** [sHR (95% CI)]	**LRTI** [sHR (95% CI)]	**UTI** [sHR (95% CI)]
**SAPS2**	**Per score point**	**1.03 (1.03 - 1.04), 0**	**0.98 (0.98 - 0.98), 0**	**1.02 (1.01 - 1.02), 0**	**1.02 (1 - 1.03), 0.01**	**1.02 (1.01 - 1.03), 0**	**1.01 (1 - 1.02), 0.01**
**Grouped,** Reference Group: 0-30	31-40	1.51 (1.14 - 1.99), 0	0.81 (0.75 - 0.88), 0	1.31 (0.92 - 1.86), 0.14	1.99 (0.98 - 4), 0.06	1.05 (0.64 - 1.72), 0.84	1.5 (0.78 - 2.88), 0.23
	41-50	2.36 (1.8 - 1.99), 0	0.69 (0.64 - 0.88), 0	1.09 (0.73 - 1.86), 0.69	1.21 (0.52 - 4), 0.66	1.12 (0.67 - 1.72), 0.66	1.18 (0.55 - 2.88), 0.67
	>50	4.08 (3.24 - 1.99), 0	0.47 (0.43 - 0.88), 0	2.31 (1.71 - 1.86), 0	2 (1.02 - 4), 0.04	2.3 (1.55 - 1.72), 0	2.19 (1.22 - 2.88), 0.01
**APACHE2**	**Per score point**	**1.06 (1.05 - 1.06), 0**	**0.97 (0.97 - 0.97), 0**	**1.03 (1.02 - 1.04), 0**	**1.02 (1 - 1.05), 0.11**	**1.03 (1.01 - 1.05), 0**	**1.03 (1.01 - 1.06), 0**
**Grouped,** Reference Group 0-10	11-20	1.48 (1.12 - 1.95), 0.01	0.83 (0.78 - 0.9), 0	1.24 (0.86 - 1.77), 0.25	2.07 (0.91 - 4.73), 0.08	0.92 (0.58 - 1.45), 0.71	2.26 (1 - 5.14), 0.05
	21-25	2.32 (1.72 - 1.95), 0	0.65 (0.6 - 0.9), 0	1.73 (1.16 - 1.77), 0.01	2.36 (0.94 - 4.73), 0.07	1.34 (0.8 - 1.45), 0.27	3.35 (1.4 - 5.14), 0.01
	>26	3.74 (2.85 - 1.95), 0	0.49 (0.45 - 0.9), 0	2.12 (1.47 - 1.77), 0	2.22 (0.92 - 4.73), 0.08	1.88 (1.19 - 1.45), 0.01	3.04 (1.3 - 5.14), 0.01
**SOFA**	**Per score point**	**1.14 (1.12 - 1.16), 0**	**0.92 (0.91 - 0.93), 0**	**1.09 (1.06 - 1.12), 0**	**1.09 (1.04 - 1.14), 0**	**1.1 (1.06 - 1.13), 0**	**1.07 (1.02 - 1.12), 0**
**Grouped,** Reference Group 0-3	4-5	1.8 (1.37 - 2.35), 0	0.79 (0.73 - 0.86), 0	1.25 (0.85 - 1.84), 0.25	2.19 (0.99 - 4.85), 0.05	1.09 (0.65 - 1.83), 0.76	1.13 (0.53 - 2.42), 0.75

	6-10	2.45 (1.95 - 2.35), 0	0.6 (0.56 - 0.86), 0	1.91 (1.4 - 1.84), 0	2.26 (1.11 - 4.85), 0.02	1.85 (1.23 - 1.83), 0	2.22 (1.25 - 2.42), 0.01
	>10	4.67 (3.67 - 2.35), 0	0.39 (0.35 - 0.86), 0	2.79 (1.97 - 1.84), 0	3.21 (1.49 - 4.85), 0	2.82 (1.79 - 1.83), 0	2.31 (1.14 - 2.42), 0.02
**Core-10-TISS**	**Per score point**	**1.06 (1.05 - 1.07), 0**	**0.96 (0.96 - 0.97), 0**	**1.04 (1.03 - 1.05), 0**	**1.06 (1.04 - 1.08), 0**	**1.04 (1.02 - 1.05), 0**	**1.03 (1 - 1.05), 0.02**
**Grouped,** Reference Group 0-20	21-30	1.34 (1.02 - 1.77), 0.04	0.8 (0.74 - 0.86), 0	2.13 (1.37 - 3.31), 0	2.16 (0.71 - 6.51), 0.17	2.07 (1.19 - 3.6), 0.01	1.48 (0.73 - 3.01), 0.28
	31-35	2.04 (1.53 - 1.77), 0	0.6 (0.55 - 0.86), 0	2.92 (1.85 - 3.31), 0	5.51 (1.85 - 6.51), 0	2.26 (1.25 - 3.6), 0.01	1.6 (0.73 - 3.01), 0.24
	>35	3.88 (2.98 - 1.77), 0	0.41 (0.38 - 0.86), 0	3.48 (2.23 - 3.31), 0	5.98 (2.05 - 6.51), 0	2.55 (1.43 - 3.6), 0	2.37 (1.15 - 3.01), 0.02

Legend: sHR, subdistribution hazard ratio; CI, confidence interval; SAPS2, Simplified Acute Physiology Score II; Core-10-TISS, Simplified Therapeutic Intervention Scoring System; APACHE2, Acute Physiology And Chronic Health Evaluation II; SOFA, Sequential Organ Failure Assessment

*Significance level: ***<0.001, **<0.01, * <0.05*

All models are adjusted by sex, admission from external hospital, and ICU type.

Each score was evaluated once as a continuous variable ("per score point", bold) and grouped.
