# Supplementary material for: SAPS2, APACHE2, SOFA, and Core-10-TISS upon admission as risk indicators for ICU-acquired infections: a retrospective cohort study
Source: Infection. 2023 Jan 13;51(4):993–1001. doi: 10.1007/s15010-022-01972-y (PMC10352405; doi:10.1007/s15010-022-01972-y)
Supplement: Supplementary file 4 — Baseline characteristics of 5,053 ICU admissions, stratified by the occurrence of an ICU-acquired infection and all competing events (PDF 42 KB) [file 15010_2022_1972_MOESM4_ESM.pdf]

SAPS2, APACHE2, SOFA, and Core-10-TISS upon admission as risk indicators for ICU-acquired infections: a retrospective cohort study
published in: Infection

Authors: Katharina Ginter[1], Frank Schwab[1], Michael Behnke[1], Martin Wolkewitz[2], Petra Gastmeier[1], Christine Geffers[1,3] and Friederike Maechler[1,3]*

[1]Institute of Hygiene and Environmental Medicine, Charité - Universitätsmedizin Berlin, corporate member of Freie Universität Berlin, Humboldt-Universität zu Berlin, and Berlin Institute of Health, Campus Benjamin Franklin, Hindenburgdamm 27, 12203 Berlin, Germany

[2] Institute of Medical Biometry and Statistics, Faculty of Medicine and Medical Center, University of Freiburg, Stefan Meier Str. 26, 79104 Freiburg, Germany

[*]corresponding author, correspondence: friederike.maechler@charite.de

[3]these authors contributed equally and share last authorship

Additional File 4: Baseline characteristics of 5,053 ICU admissions, stratified by the occurrence of an ICU-acquired infection and all competing events

Supplementary Table 7: Baseline characteristics of 5,053 ICU admissions, stratified by the occurrence of an ICU-acquired infection and all competing events

	**Overall (N=5053)**	**Infection (N=253)**	**Discharge (N=4232)**	**Death (N=568)**
Sex Male	2983 (59.0%)	153 (60.5%)	2501 (59.1%)	329 (57.9%)
Female	2070 (41.0%)	100 (39.5%)	1731 (40.9%)	239 (42.1%)
Age (in years), Mdn [Q1-Q3]	67.0 [55.0-77.0]	68.0 [56.0-77.0]	67.0 [55.0-77.0]	72.0 [59.0-80.0]
Admission from external hospital	922 (18.2%)	56.0 (22.1%)	739 (17.5%)	127 (22.4%)
Deceased during ICU stay	631 (12.5%)	63.0 (24.9%)	0 (0%)	568 (100%)
Length of stay (in days), Mdn [Q1-Q3]	6.00 [4.00-12.0]	27.0 [17.0-43.0]	5.00 [4.00-10.0]	8.00 [4.00-14.0]
Ventilation on admission day	3249 (64.3%)	225 (88.9%)	2517 (59.5%)	507 (89.3%)
Type of ICU medical	2063 (40.8%)	113 (44.7%)	1644 (38.8%)	306 (53.9%)
surgical	2990 (59.2%)	140 (55.3%)	2588 (61.2%)	262 (46.1%)
**Scoring Systems**				
SAPS2, Mdn [Q1-Q3]	38.0 [27.0-52.0]	44.0 [32.0-61.0]	36.0 [26.0-49.0]	51.0 [37.0-65.0]
APACHE2, Mdn [Q1-Q3]	17.0 [11.0-24.0]	21.0 [14.0-27.0]	16.0 [11.0-23.0]	23.0 [16.0-30.0]
Core-10-TISS, Mdn [Q1-Q3]	28.0 [22.0-34.0]	31.0 [25.0-36.0]	28.0 [22.0-32.0]	33.0 [26.0-40.0]
SOFA, Mdn [Q1-Q3]	5.00 [3.00-8.00]	7.00 [4.00-10.0]	5.00 [2.00-7.00]	8.00 [4.75-11.0]
**ICU-acquired infections**				
Time until first infection (in days), Mdn [Q1-Q3]	11.0 [6.00-22.0]	11.0 [6.00-22.0]		
PBSI	54.0 (1.1%)	54.0 (21.3%)		
LRTI	144 (2.8%)	144 (56.9%)		
UTI	70.0 (1.4%)	70.0 (27.7%)		
Incidence density per SAPS2 quartile (ICU-acquired infections per 1000 hospital days)				
Q1 (<27)	4.131			
Q2 (27-38)	4.530			
Q3 (39-52)	3.692			
Q4 (>52)	6.001			

	Overall (N=5053)	Infection (N=253)	Discharge (N=4232)	Death (N=568)
**Comorbidities / Charlson Comorbidity Index (CCI)**				
Missing values	519 (10.3%)	13.0 (5.1%)	443 (10.5%)	63.0 (11.1%)
CCI, Mdn [Q1-Q3]	6.00 [4.00-9.00]	7.00 [4.00-9.00]	6.00 [4.00-9.00]	8.00 [6.00-10.0]
Myocardial infarction	141 (2.8%)	9.00 (3.6%)	116 (2.7%)	16.0 (2.8%)
Congestive heart failure	1056 (20.9%)	71.0 (28.1%)	804 (19.0%)	181 (31.9%)
Peripheral vascular disease	630 (12.5%)	42.0 (16.6%)	497 (11.7%)	91.0 (16.0%)
Cerebrovascular disease	911 (18.0%)	61.0 (24.1%)	717 (16.9%)	133 (23.4%)
Dementia	217 (4.3%)	7.00 (2.8%)	178 (4.2%)	32.0 (5.6%)
Chronic lung disease	979 (19.4%)	77.0 (30.4%)	792 (18.7%)	110 (19.4%)
Rheumatism	117 (2.3%)	8.00 (3.2%)	91.0 (2.2%)	18.0 (3.2%)
Peptic ulcer	165 (3.3%)	14.0 (5.5%)	136 (3.2%)	15.0 (2.6%)
Mild liver disease	422 (8.4%)	35.0 (13.8%)	336 (7.9%)	51.0 (9.0%)
Moderate to severe liver disease	373 (7.4%)	36.0 (14.2%)	209 (4.9%)	128 (22.5%)
Diabetes without complications	865 (17.1%)	45.0 (17.8%)	718 (17.0%)	102 (18.0%)
Diabetes with chronic complications	247 (4.9%)	14.0 (5.5%)	203 (4.8%)	30.0 (5.3%)
Renal disease	2132 (42.2%)	136 (53.8%)	1651 (39.0%)	345 (60.7%)
Cancer	1255 (24.8%)	59.0 (23.3%)	1065 (25.2%)	131 (23.1%)
Metastatic cancer	633 (12.5%)	20.0 (7.9%)	541 (12.8%)	72.0 (12.7%)
Leukemia	47.0 (0.9%)	6.00 (2.4%)	26.0 (0.6%)	15.0 (2.6%)
Lymphoma	99.0 (2.0%)	5.00 (2.0%)	77.0 (1.8%)	17.0 (3.0%)
Hemiplegia	570 (11.3%)	29.0 (11.5%)	482 (11.4%)	59.0 (10.4%)
HIV/AIDS	11.0 (0.2%)	1.00 (0.4%)	8.00 (0.2%)	2.00 (0.4%)
Any type of infection (CCI)	3041 (60.2%)	223 (88.1%)	2405 (56.8%)	413 (72.7%)
Any sepsis (CCI)	1611 (31.9%)	151 (59.7%)	1151 (27.2%)	309 (54.4%)
Any Pneumonia (CCI)	1607 (31.8%)	167 (66.0%)	1226 (29.0%)	214 (37.7%)

Infection, ICU-acquired infection; Death, In-hospital death without prior ICU-acquired infection;
Discharge, Discharge without prior ICU-acquired infection or in-hospital death
Abbreviations: N, Number; SAPS2, Simplified Acute Physiology Score II; Core-10-TISS, Simplified Therapeutic Intervention Scoring System; APACHE2, Acute Physiology And Chronic Health Evaluation II; SOFA, Sequential Organ Failure Assessment; AIDS, Acquired immune deficiency syndrome; HIV, Human immunodeficiency virus; PBSI, Primary bloodstream infection; LRTI, Lower respiratory tract infection (pneumonia and/or bronchitis); UTI, Urinary tract infection; Q1, First Quartile; Q3, Third Quartile; Mdn, Median
