# Supplementary material for: SAPS2, APACHE2, SOFA, and Core-10-TISS upon admission as risk indicators for ICU-acquired infections: a retrospective cohort study
Source: Infection. 2023 Jan 13;51(4):993–1001. doi: 10.1007/s15010-022-01972-y (PMC10352405; doi:10.1007/s15010-022-01972-y)
Supplement: Supplementary file 5 — Results of the cause-specific Cox models for the scores SAPS2, APACHE, SOFA and Core-10-TISS (PDF 53 KB) [file 15010_2022_1972_MOESM5_ESM.pdf]

SAPS2, APACHE2, SOFA, and Core-10-TISS upon admission as risk indicators for ICU-acquired infections: a retrospective cohort study

published in: Infection

Authors: Katharina Ginter[1], Frank Schwab[1], Michael Behnke[1], Martin Wolkewitz[2], Petra Gastmeier[1], Christine Geffers[1,3] and Friederike Maechler[1,3]*

[1]Institute of Hygiene and Environmental Medicine, Charité - Universitätsmedizin Berlin, corporate member of Freie Universität Berlin, Humboldt-Universität zu Berlin, and Berlin Institute of Health, Campus Benjamin Franklin, Hindenburgdamm 27, 12203 Berlin, Germany

[2] Institute of Medical Biometry and Statistics, Faculty of Medicine and Medical Center, University of Freiburg, Stefan Meier Str. 26, 79104 Freiburg, Germany

[*]corresponding author, correspondence: friederike.maechler@charite.de

[3]these authors contributed equally and share last authorship

Additional File 5: Results of the cause-specific Cox models for the scores SAPS2, APACHE, SOFA and Core-10-TISS

Supplementary Table 8: Results of the cause-specific Cox models for the scores SAPS2, APACHE, SOFA and Core-10-TISS

		**Death** [HR (95% CI)]	**Discharge** [HR (95% CI)]	**ICU-acquired infection** [HR (95% CI)]	**ICU-acquired infection: Type**		
					**PBSI** [HR (95% CI)]	**LRTI** [HR (95% CI)]	**UTI** [HR (95% CI)]
**SAPS2**	**Per score point**	**1.02 (1.01 - 1.02), 0.00**	**0.98 (0.98 - 0.98), 0.00**	**1.00 (1.00 - 1.01), 0.34**	**1.00 (0.99 - 1.01), 0.89**	**1.01 (1.00 - 1.02), 0.14**	**1.00 (0.98- 1.01), 0.54**
**Grouped,** Reference Group: 0-30	31-40	1.27 (0.94 - 1.72), 0.12	0.81 (0.75 - 0.88), 0	1.18 (0.81 - 1.7), 0.39	2.32 (1.04 - 5.2), 0.04	0.92 (0.55 - 1.52), 0.74	1.12 (0.56- 2.25), 0.74
	41-50	1.89 (1.42 - 2.51), 0	0.69 (0.63 - 0.75), 0	0.74 (0.47 - 1.14), 0.17	1.29 (0.49 - 3.35), 0.6	0.76 (0.44 - 1.34), 0.35	0.67 (0.28- 1.56), 0.35
	>50	2.44 (1.91 - 3.13), 0	0.47 (0.44 - 0.51), 0	1.26 (0.91 - 1.74), 0.17	1.22 (0.55 - 2.7), 0.62	1.29 (0.85 - 1.96), 0.24	1.04 (0.56- 1.91), 0.91
**APACHE2**	**Per score point**	**1.03 (1.02 - 1.04), 0.00**	**0.97 (0.97 - 0.98), 0.00**	**1.01 (0.99 - 1.02), 0.45**	**0.99 (0.96 - 1.02), 0.42**	**1.01 (0.99 - 1.03), 0.29**	**1.01 (0.98- 1.03), 0.60**
**Grouped,** Reference Group 0-10	11-20	1.25 (0.93 - 1.67), 0.13	0.85 (0.79 - 0.92), 0	0.99 (0.68 - 1.45), 0.97	1.64 (0.66 - 4.05), 0.28	0.78 (0.49 - 1.26), 0.31	1.55 (0.67- 3.59), 0.30
	21-25	1.63 (1.18 - 2.23), 0	0.68 (0.61 - 0.75), 0	1.25 (0.82 - 1.9), 0.3	1.96 (0.74 - 5.16), 0.18	0.91 (0.53 - 1.56), 0.72	2.02 (0.83- 4.93), 0.12
	>26	2.17 (1.64 - 2.88), 0	0.49 (0.44 - 0.54), 0	1.04 (0.71 - 1.54), 0.84	0.93 (0.35 - 2.49), 0.88	1.04 (0.64 - 1.67), 0.89	1.36 (0.58- 3.23), 0.48
**SOFA**	**Per score point**	**1.06 (1.04 - 1.08), 0.00**	**0.91 (0.91 - 0.92), 0.00**	**1.00 (0.98 - 1.03), 0.74**	**0.99 (0.93 - 1.05), 0.78**	**1.03 (0.99 - 1.07), 0.18**	**0.99 (0.93 - 1.05), 0.78**
**Grouped,** Reference Group 0-3	4-5	1.6 (1.21 - 2.13), 0	0.8 (0.73 - 0.87), 0	0.87 (0.58 - 1.31), 0.51	1.08 (0.45 - 2.61), 0.86	0.91 (0.53 - 1.57), 0.74	1.08 (0.45 - 2.61), 0.86
	6-10	1.63 (1.28 - 2.09), 0	0.59 (0.54 - 0.63), 0	1.04 (0.75 - 1.44), 0.82	1.06 (0.51 - 2.22), 0.87	1.13 (0.73 - 1.75), 0.57	1.06 (0.51 - 2.22), 0.87

	>10	2.14 (1.65 - 2.79), 0	0.36 (0.32 - 0.4), 0	1.02 (0.71 - 1.48), 0.91	0.89 (0.39 - 2.03), 0.79	1.29 (0.79 - 2.09), 0.31	0.89 (0.39 - 2.03), 0.79
**Core-10-TISS**	**Per score point**	**1.03 (1.02 - 1.04), 0.00**	**0.96 (0.96 - 0.97), 0.00**	**1.00 (0.99 - 1.02), 0.59**	**1.01 (0.98 - 1.04), 0.41**	**1.00 (0.99 - 1.02), 0.67**	**0.97 (0.92- 1.02), 0.23**
**Grouped,** Reference Group 0-20	21-30	1.17 (0.88 - 1.56), 0.28	0.83 (0.76 - 0.9), 0	1.68 (1.07 - 2.64), 0.02	1.59 (0.53 - 4.79), 0.41	1.51 (0.86 - 2.63), 0.15	0.85 (0.38- 1.87), 0.68
	31-35	1.16 (0.86 - 1.58), 0.33	0.56 (0.51 - 0.61), 0	1.51 (0.94 - 2.42), 0.09	2.29 (0.77 - 6.86), 0.14	1.25 (0.69 - 2.27), 0.46	1.02 (0.55- 1.88), 0.96
	>35	1.91 (1.44 - 2.52), 0	0.41 (0.37 - 0.45), 0	1.33 (0.83 - 2.12), 0.24	1.74 (0.58 - 5.19), 0.32	1.08 (0.6 - 1.96), 0.79	0.76 (0.37- 1.56), 0.46

Legend: HR, hazard ratio; CI, confidence interval; SAPS2, Simplified Acute Physiology Score II; Core-10-TISS, Simplified Therapeutic Intervention Scoring System; APACHE2, Acute Physiology And Chronic Health Evaluation II; SOFA, Sequential Organ Failure Assessment
*Significance level: ***<0.001, **<0.01, * <0.05*
All models are adjusted by sex, admission from external hospital, and ICU type.
Each score was evaluated once as a continuous variable ("per score point", bold) and grouped.
